# Comparative Evaluation of Fracture Resistance of Carbon Fiber Posts and Glass Fiber Posts in Permanent Anterior Teeth: An In Vitro Study

**DOI:** 10.7759/cureus.60647

**Published:** 2024-05-20

**Authors:** Saurabh Joshi, Pratima Shah, Dhananjay Gandhage, Viddyasagar Mopagar, Rajesh Krishna Malge, Gowri Pendyala

**Affiliations:** 1 Department of Pediatric Dentistry, Rural Dental College, Pravara Institute of Medical Sciences, Loni, IND; 2 Department of Prosthodontics, Crown and Bridge, and Implantology, Dr. D. Y. Patil Dental College & Hospital, Dr. D. Y. Patil Vidyapeeth, Pune, IND; 3 Department of Pedodontics and Preventive Dentistry, Rural Dental College, Pravara Institute of Medical Sciences, Loni, IND; 4 Department of Pedodontics and Preventive Dentistry, ESIC (Employee's State Insurance Corporation) Dental College, Kalaburagi, IND; 5 Department of Periodontics, Rural Dental College, Pravara Institute of Medical Sciences, Loni, IND

**Keywords:** resistance, fracture, carbon fibre, glass fibre, fibre post

## Abstract

Introduction

Dental caries and traumatic injuries often lead to tooth loss in adolescents and adults, necessitating endodontic treatment and subsequent restoration. Restoring such teeth presents a challenge due to varying degrees of substance loss. After endodontic treatment, the choice of an appropriate post is crucial for long-term stability. While metal posts are sturdy, they lack aesthetics and may cause root fractures. Fiber posts, such as carbon and glass fiber, offer improved aesthetics and mechanical properties, but their comparative performance warrants investigation.

Materials and methods

A total of 30 extracted anterior single-rooted teeth were divided into two groups to receive either carbon fiber or glass fiber posts. After endodontic treatment and post-space preparation, the posts were cemented using a dual polymerizing adhesive resin composite. Fracture resistance was assessed using a universal testing machine.

Results

The mean fracture resistance of the carbon fiber post group was recorded at 271.2 N, whereas the glass fiber post group exhibited a significantly higher mean fracture resistance of 416.133 N. This difference in fracture resistance between the two groups was found to be statistically significant (p < 0.05).

Conclusion

Glass fiber post systems demonstrated superior fracture resistance compared to carbon fiber post systems in anterior single-rooted teeth. These findings support the clinical preference for glass fiber posts in restoring endodontically treated anterior teeth, offering both mechanical reliability and aesthetic advantages. However, further research, including long-term clinical trials, is warranted to validate these findings and assess the overall clinical performance and longevity of fiber post systems in real-world settings.

## Introduction

Dental caries and traumatic injuries to anterior teeth are etiological factors for tooth loss in adolescents and adults. Caries result when the net demineralization exceeds remineralization [1]. Trauma, in its various degrees of severity, is responsible for non-diseased tooth loss [2]. It ranges from habitual and frictional trauma due to uncontrolled factors such as accidents, falls, and trauma from occlusion [2,3]. Restoring such teeth is both an endodontic necessity and a prosthodontic challenge. When restoring endodontically treated teeth with varying degrees of substance loss, the final restoration complex must be able to withstand long-term masticatory forces. After the endodontic treatment is completed and depending on how much coronal structure remains, three types of restorations can be performed: (i) direct or indirect restorations, (ii) partial or full coverage restorations, and (iii) the placement of a post, core build-up, and an appropriate full coverage prosthesis. Currently, the latter option is more commonly practiced than the former options. The selection of an appropriate post depends on the following factors: remaining tooth structure, position of the tooth in the dental arch, need for aesthetics, and functional loading of the tooth [4]. Cast metal posts are rigid and unaesthetic. Additionally, due to the rigidity of metal posts, microfractures can occur in the roots of the anterior teeth, necessitating multiple steps in fabrication [5].

To compensate for the weakness of metal posts and improve the optical effects of aesthetic restorations, fiber posts were introduced [6]. Assif and Gorfil reported that when root canal-treated teeth were restored with posts, core stress concentration took place at the coronal region, at the interface of the different moduli of elasticity [7]. In order to minimize root weakness, carbon fiber and silica fiber reinforced posts were introduced in the last decade [8,9]. Carbon fiber posts were introduced by Reynaud and Duret in 1990. This dowel was composed of carbon fibers, 8 microns in diameter, embedded in an epoxy resin matrix. Due to their superior mechanical properties, they were found to have better fracture propagation resistance. However, carbon fiber posts were black in color and did not prove to be very aesthetic restorations when combined with ceramic crowns [9].

This led to the discovery of silica fiber posts, namely, glass fiber and quartz fiber posts. These were essentially composed of pre-stretched fibers bound by a resin matrix. Due to their excellent mechanical properties and transparent appearance, glass fiber systems gained popularity. Based on their composition, glass can be classified as S-glass (High-Strength) and E-glass (Electric) [9]. These were the most commonly used varieties of glass to fabricate glass fiber posts. Caputo and Standlee stated that posts are necessary to enable the clinician to rebuild enough tooth structure to retain restorations [10]. However, in the process of preparing post space, a significant amount of gutta-percha, root canal sealer, and dentin are lost, making the remaining structure very brittle and prone to fracture. Thus, this study was planned to evaluate the fracture resistance of two different fiber post systems: carbon fiber and glass fiber posts. Both systems have the same background matrix, which is epoxy resin, and were tested in permanent anterior single-rooted, single-canal teeth.

## Materials and methods

This comparative study was conducted at the Rural Dental College, Pravara Institute of Medical Sciences (PIMS), Loni, Maharastra, India. It was approved by the Institutional Ethical Committee, PIMS (approval number: PMT/PIMS/IEC/2022/049).

**Figure 1:**
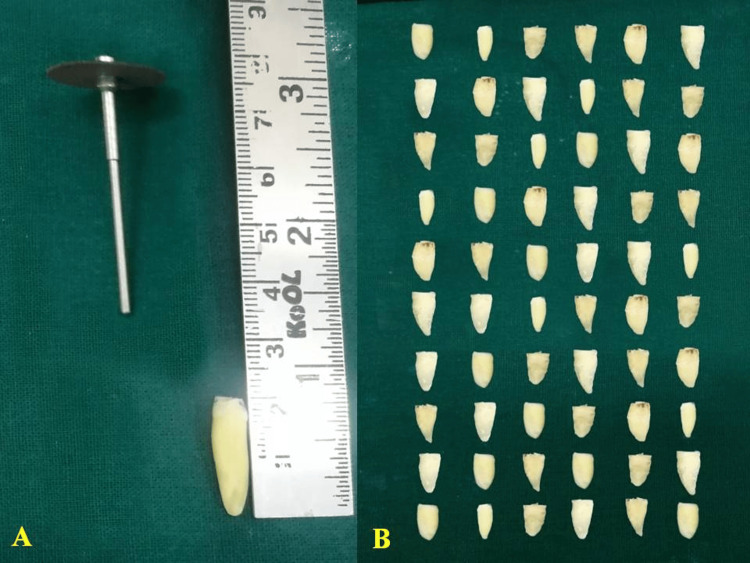
Decoronation of the teeth: (A) measuring of the extracted teeth, (B) sequencing of the teeth

In our investigation, a total of 60 extracted permanent anterior teeth were utilized, comprising incisors, canines, and premolars characterized by single roots and single canals. The teeth used in our study were either recommended for extraction for orthodontic reasons or were periodontally compromised. The collected teeth underwent meticulous cleaning procedures to eliminate any calculus or debris accumulation. Following this, they were thoroughly washed with tap water and subjected to a disinfection process involving immersion in a 2% sodium hypochlorite solution for a duration of two hours, as recommended by the manufacturer. Subsequently, autoclaving was employed to ensure operator safety, after which the teeth were preserved in a 5% formaldehyde solution (Figure 1).

**Figure 2:**
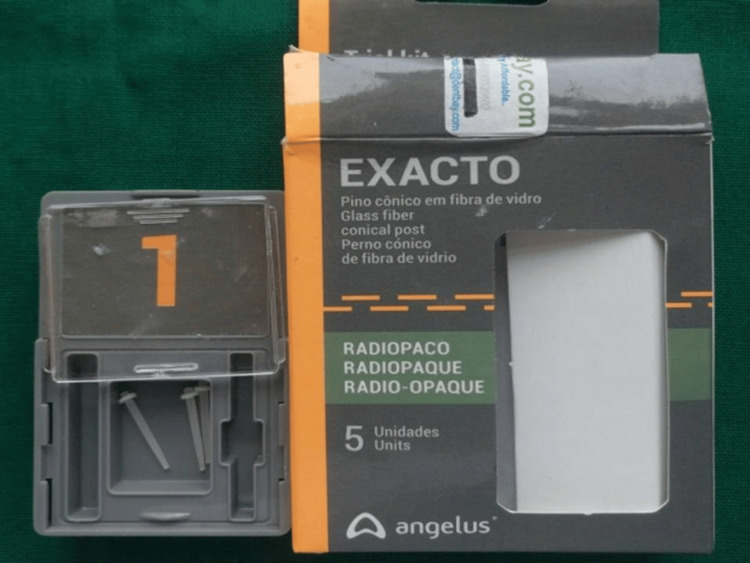
Carbon fiber post system

**Figure 3:**
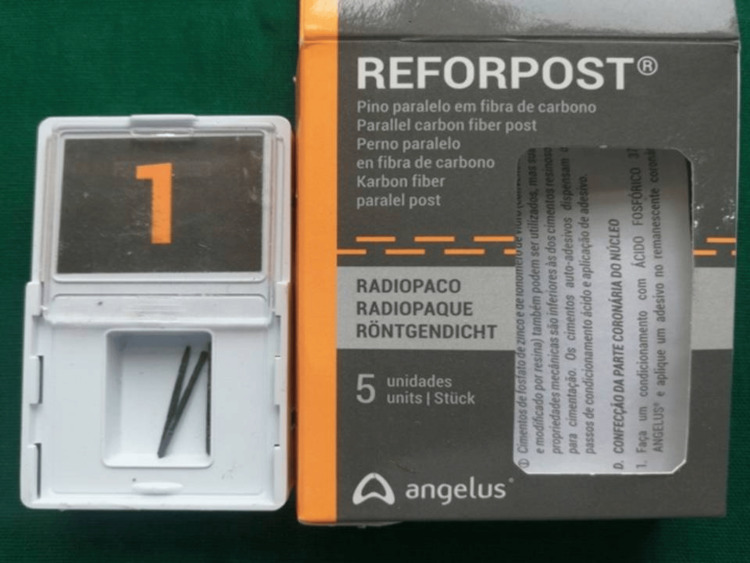
Glass fiber post systems

A total of 60 teeth, after thorough cleaning and disinfection, were randomly divided into two groups, A and B, to receive two different fiber posts, post endodontic treatment. Group A consisted of 30 permanent maxillary or mandibular single-rooted anterior teeth, which were prepared to receive carbon fiber posts (Exacto; Angelus Indústria de Produtos Odontológicos S/A, Lodrina, Brazil) (Figure 2) and Group B consisted of 30 permanent maxillary or mandibular, single-rooted, anterior teeth which were prepared to receive glass fiber posts (Reforpost; Angelus Indústria de Produtos Odontológicos S/A) (Figure 3).

Preparation of the sample

Endodontic Treatment

A number 2, round carbide bur (SS White Surgical Length; SS White Dental, New Jersey, United States) was used to prepare the access cavity. The root canal permeability was checked with a # 10 K file (Mani Inc., Takenzawa, Japan). The teeth were instrumented till # 20 K file and further biomechanical preparation was done by ProTaper rotary files (Dentsply Sirona Inc., Charlotte, North Carolina, United States) up to F3 file 1 mm from the apex (Figure 6). The teeth were irrigated with 5.25% sodium hypochlorite (Prime Dental Products Pvt. Ltd, Thane, Maharastra, India) between each instrumentation. A final flush was given with 17% ethylenediamine tetraacetic acid (EDTA) (Prime Dental Products Pvt. Ltd) and final irrigation was done with 5.25% sodium hypochlorite (Prime Dental Products Pvt. Ltd). Teeth were dried with ProTaper Paper Points (Dentsply Sirona Inc.) and obturated with ProTaper GP points (6%) (Dentsply Sirona Inc.).The obturation was carried out by lateral condensation, and condensing 2% ProTaper GP points into the lateral canals.

Standardization of the Posts

Posts that were most similar in size were taken for the study. The length of the glass fibers was 15 mm whereas the length of the carbon system was around 19-20 mm. Each of the fibers was cut so their length would cover two-thirds of the root canal system. The diameter chosen for each post was 1.5 mm.

Preparation of Post Space

**Figure 4:**
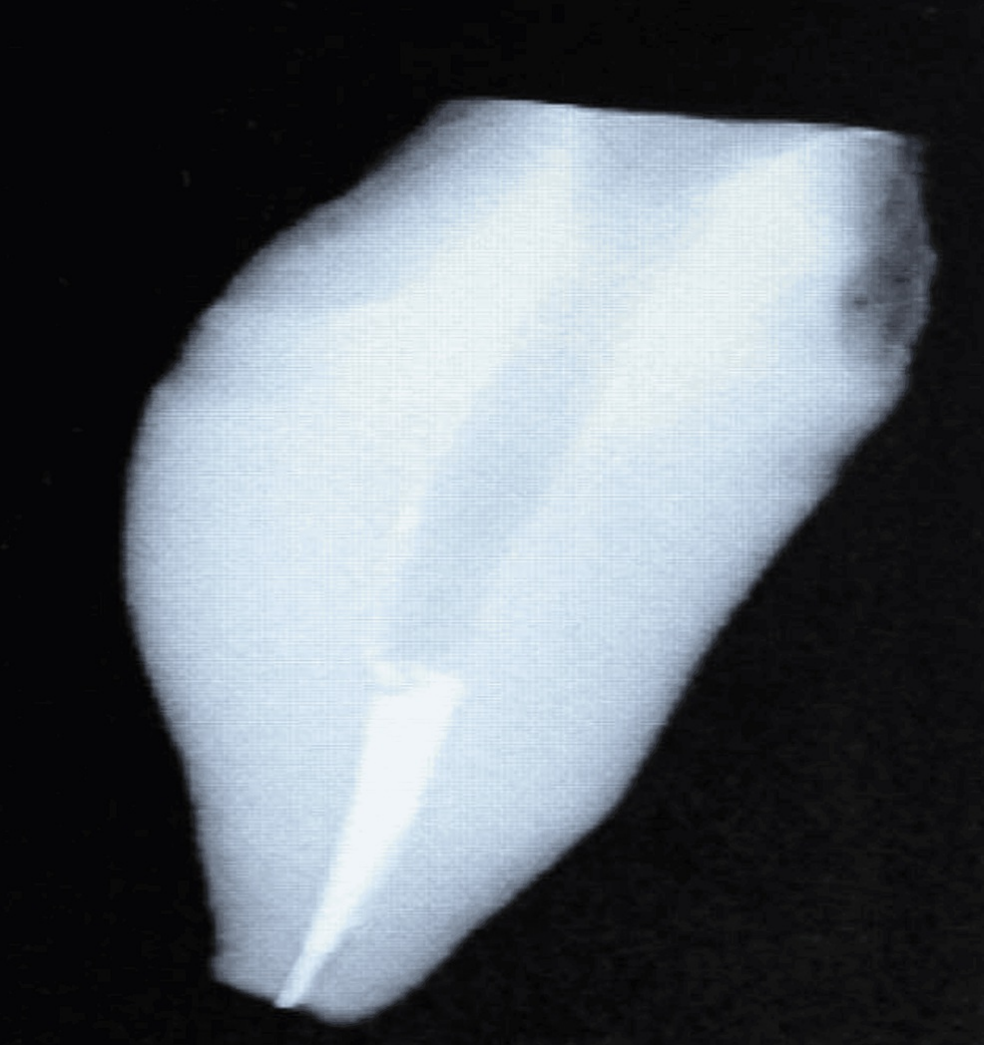
Post space preparation

Exacto and Reforpost: The entrance of the root canal was widened leaving a 1.25 mm thick wall to a depth of 4 mm. The rest of the root was unsealed to the depth of 8-10 mm leaving about 3-6 mm, using Peeso reamers (Dentsply Sirona Inc.). The sizes used were #0, #1, and #2 (or #3). The accompanying manufacturer-recommended twist drills were used for the final preparation passively. A carbon fiber post and glass fiber posts of the diameter 1.5 mm were selected to be cemented in with dual polymerizing adhesive resin composite (LuxaCore Z Dual Smartmix A3; DMG Chemisch-Pharmazeutische Fabrik GmbH, Hamburg, Germany) (Figure 4).

Cementation of the Posts

**Figure 5:**
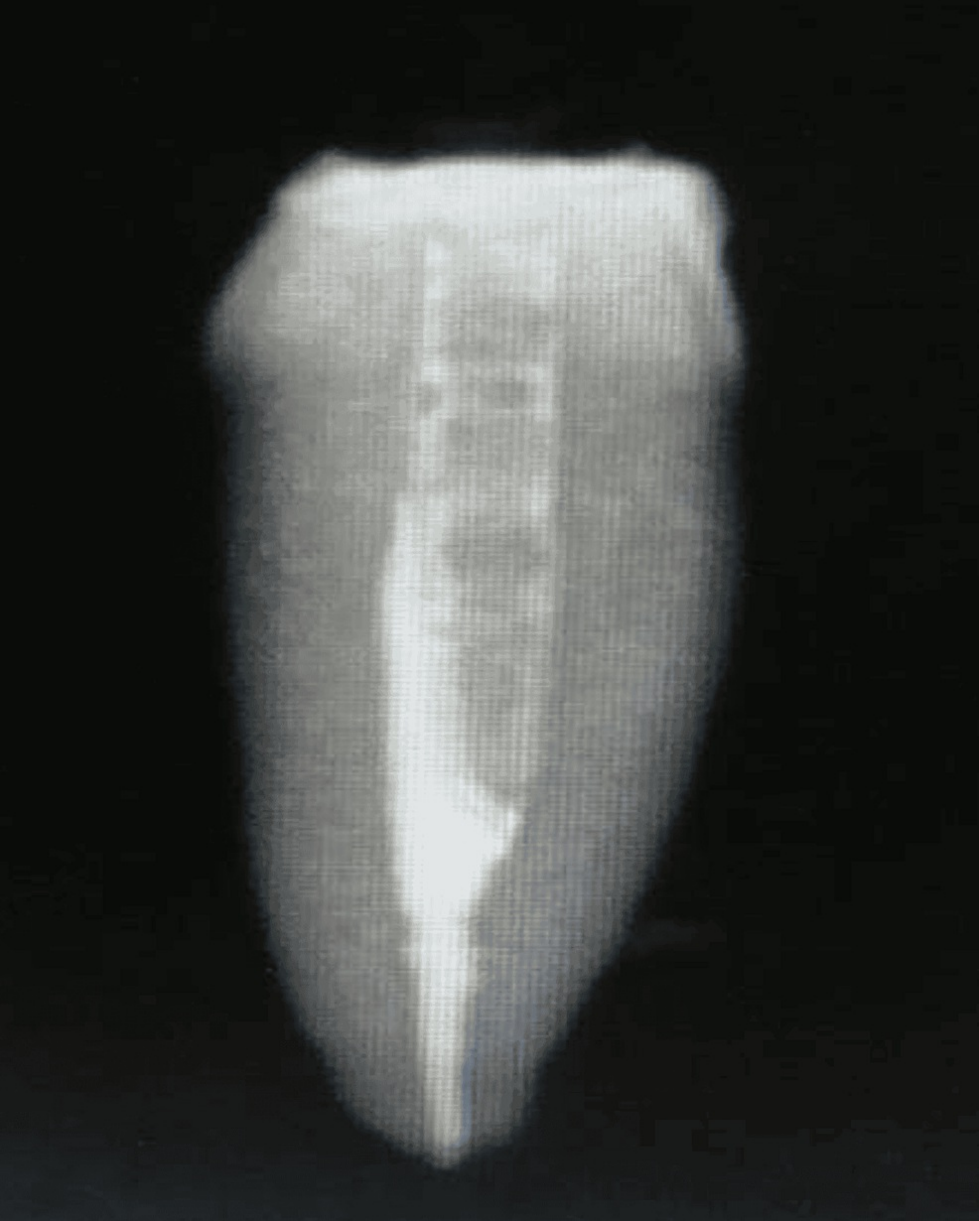
Post cementation

Interradicular dentin was etched with 35% phosphoric acid (Actino Gel; Prevest DenPro Limited, Bari Brahmana, Jammu, India) for 15 seconds and washed with water spray. Two consecutive coats of Single Bond adhesive (3M Company, Saint Paul, Minnesota, United States) were applied to the canals. This was left to dry for five seconds and the excess was removed using paper points. The adhesive was light-cured for 10 seconds. The adhesive cement was mixed for 10 seconds and a thin coat was applied to the dentinal walls using a periodontal probe. Another thin layer was applied to each of the posts and the post was inserted into the canal. Excess cement was removed and the remainder was light-cured for 40 seconds (Figure 5). The curing light (LY-B200) that was used had a wavelength of 420-480 nm and an intensity of 1200-2000 mw/cm². Its curing depth was 3 mm/5 seconds.

Mounting the Samples and Simulating the Periodontal Ligament

A thin layer of modeling wax was applied on the root surface of each tooth. Then each tooth was embedded into acrylic blocks and removed immediately before the setting of acrylic. Color coding was done for each group so as to differentiate and to facilitate the ease of identification. Then all the specimens were put into a dewaxing bath for five minutes with only the root surfaces being immersed in the bath. Then the root-shaped well in each acrylic block was coated with a layer of aqua gel (Ultrasun Aqua Gel; Swisskem Healthcare, Himachal Pradesh, India), and the teeth were re-immersed into the blocks. Thus, the space created by wax was occupied by aqua gel and resembled the periodontal ligament (PDL) space of each tooth.

Checking for Fracture Resistance

A TUF-C-1000 Servo Universal Testing Machine (Fine Spavy Associates & Engineers Private Limited, Miraj, Maharastra, India) applied controlled loads using an acrylic mounting block designed to stabilize specimens. A compressive head with a steel ball-end (0.5 mm diameter) was angled at 50 degrees to produce a 130-degree angle between the long axis of the tooth specimen and the compressive head. Load application mimicked inter-incisal forces encountered in the mouth, with displacement and load magnitude recorded using a compressive load cell. Failure threshold was determined when load increment was no longer feasible, with results quantified in Newtons.

Statistical analysis

The gathered data underwent recording and entry into a Microsoft Excel spreadsheet (Microsoft Corporation; Redmond, Washington, United States). Statistical analysis was performed utilizing IBM SPSS Statistics for Windows, Version 20.0 (Released 2011; IBM Corp., Armonk, New York, United States). The normality of variables was assessed employing the Kolmogorov-Smirnov test. Descriptive statistics were computed, including mean values and standard deviation. Independent t-tests were employed for intra-group comparisons at a significance level of P ≤ 0.05.

## Results

Table 1 illustrates the comprehensive descriptive statistics comparing the mean values and standard deviations of critical mechanical parameters between carbon fiber posts and their glass fiber counterparts. The mean values for carbon fiber posts and glass fiber posts are meticulously measured at 271.2 N and 416.1333 N, respectively, showcasing intrinsic differences in load-bearing capabilities. Furthermore, the standard deviations, serving as indicators of dispersion within the datasets, stand at 51.0338 N and 22.6143 N for carbon fiber and glass fiber posts, respectively.

**Table 1:** Comparison of mean values and standard deviations

Groups	Carbon Fiber Posts	Glass Fiber Posts
Mean	271.2	416.1333
Standard Deviation	51.0338	22.6143
Minimum Value (Newtons)	198	390
Maximum Value (Newtons)	340	445

Delving deeper into the mechanical spectrum, the analysis extends to encompass the minimum and maximum values, which is crucial for delineating the range of mechanical behavior exhibited by each material variant. For carbon fiber posts, the minimum and maximum values stand at 198 and 340 N, respectively, elucidating the span within which mechanical forces can be withstood. Conversely, glass fiber posts manifest a marginally higher range, characterized by minimum and maximum values of 390 N and 445 N, respectively.

The subsequent scrutiny via intra-group comparison utilizing the venerable Student's t-test (Table 2) unveils profound disparities between the two material cohorts. When juxtaposing the mechanical attributes of other groups against the benchmark set by carbon fiber posts, a striking p-value of <0.0000001 emerges, accompanied by a mean difference of -144.933 N for glass fiber posts. This empirical evidence underscores a palpable divergence in load-bearing capacity, with glass fiber posts exhibiting a discernibly lower mean value compared to their carbon counterparts. The obtained p-value, attaining statistical significance, accentuates the robustness and validity of the findings.

**Table 2:** Comparison of the fracture resistance of glass fiber post and carbon fiber posts *Pearson's coefficient was used;  p<0.005 is significant

Groups	p-values*	Mean difference
Carbon fiber posts Vs. Glass fiber posts	<0.0000001	-144.933

These findings substantiate the pivotal role of material selection in the domain of restorative dentistry, ascertaining carbon fiber posts as exemplars of superior mechanical performance vis-à-vis glass fiber counterparts. Such meticulous analyses are instrumental for clinicians and researchers alike, offering invaluable insights for optimizing treatment modalities and material choices, thereby augmenting the efficacy and longevity of dental restorations.

## Discussion

The utilization of fiber posts for the rehabilitation of endodontically treated teeth is experiencing a notable surge in acceptance within the dental community. This upward trajectory in adoption is largely credited to substantial advancements in our comprehension and foresight regarding the intricacies involved in dentin bonding processes [10]. An improved understanding of the various modes of fiber reinforcement has given clinicians diverse options for bolstering lost tooth structure subsequent to initial non-surgical endodontic therapy and retreatment procedures [11]. Within these alternatives, the utilization of pre-fabricated, high-quality fiber posts stands out for their convenience in handling, concurrently eliminating potential flaws that might be inadvertently introduced during the on-site fabrication of other root reinforcement modalities [11].

Anterior teeth are particularly susceptible to angular and shear forces, necessitating post-supported full coverage restorations more frequently compared to posterior teeth [9]. In the current study, the teeth were decoronated to a level 1 mm above the cementoenamel junction (CEJ) to replicate a clinical scenario indicative of extensive horizontal loss of tooth structure [12]. Subsequently, endodontic treatment was administered, and obturation was carried out using #30 6% GP cones and #25 2% GP cones via the lateral condensation technique.

In our investigation, we employed four distinct fiber posts with varying compositions. Group A used Exacto**,** comprising an epoxy resin matrix infused with longitudinally aligned unidirectional carbon fibers. These fibers, measuring 8 microns in diameter, constituted 64% of the dowel and were pre-stretched prior to embedding in the matrix to enhance their mechanical properties. Group B utilized Reforpost, which incorporated unidirectional glass fibers (60%) embedded within an epoxy resin matrix. These fibers were closely arranged to bolster mechanical strength, and the post exhibited a double taper design to facilitate optimal adaptation to the root canal walls. Moreover, the longitudinal alignment of fibers facilitated ease of removal in cases requiring retreatment.

Each of the selected fiber types exhibits mechanical characteristics akin to dentin and facilitates uniform stress distribution. For instance, fiber-reinforced post systems typically possess a modulus of elasticity around 20 GPa, closely resembling that of dentin, which stands at approximately 18 GPa [13]. Metal and ceramic posts are characterized as isotropic, meaning their elastic modulus remains constant regardless of the angle of insertion. In contrast, fiber posts are considered anisotropic, exhibiting varying moduli of elasticity depending on the angle of insertion. This distinction underscores the unique mechanical behavior of fiber posts compared to metal counterparts [13,14]. Fiber posts typically demonstrate their highest elastic modulus when subjected to vertical loads between 30-40 degrees, which closely mimics the forces encountered during mastication. Additionally, except carbon fiber posts, fiber posts possess a natural translucency that meets the aesthetic requirements of anterior restorations. This characteristic addresses the shortcomings commonly associated with ceramic and zirconium post systems, making fiber posts a preferred choice for anterior restoration complexities [14]. In case re-treatment is indicated, fiber posts can easily be retrieved with minimal loss of dentin, thus providing the dentist with further options.

In our investigation, load testing was conducted using the TUFC 1000 Universal Testing Machine. Analysis during specimen loading revealed that primary failure was indicated by a noticeable decline in the curve's values. This decline corresponded with visible instances of core separation or failure of individual components within the restoration complex. The mean maximum load recorded at the point of fracture was 271 N for carbon fiber posts and 416.133 N for glass fiber posts. These findings unequivocally demonstrate that carbon fiber posts exhibited lesser resistance to fracture compared to glass fiber posts.

In a study conducted by Sharma et al., wherein quartz, glass, and carbon fiber posts were compared, the quartz fiber post group exhibited the highest mean value for fracture resistance at 1318.1 MPa, followed by the glass fiber post group at 1282.6 N [15]. The carbon fiber group displayed the lowest fracture resistance with a recorded value of 1275 N. These findings align closely with our study results, where carbon fiber posts demonstrated inferior fracture resistance compared to glass fiber posts.

Utilizing in vitro testing methodologies serves as a means to evaluate effectiveness; however, these tests are inherently limited as they do not fully replicate the dynamic intraoral environment [16]. Furthermore, they lack comprehensive insights into the behavior of the tooth restoration complex prior to failure. Despite these limitations, the standardized test standards and conditions enable the uniform assessment of all parameters. It's noteworthy that while complete crown coverage was not provided, both cementation and core buildup were conducted using the same dual-cure composite material

## Conclusions

Our study revealed that carbon fiber post systems exhibited inferior resistance to fracture compared to glass fiber posts, highlighting potential limitations in their clinical application. To substantiate these findings conclusively, further investigations are warranted. Additional in vitro studies are essential to comprehensively examine the mechanical properties and behavior of both carbon and glass fiber posts, scrutinizing factors such as load distribution and failure mechanisms. In vivo experiments on live subjects or animal models and clinical trials involving actual patient cases are imperative for validating our findings in real-world scenarios and assessing factors like longevity and biocompatibility. Through these meticulous investigations, we aim to enhance our understanding of how carbon and glass fiber posts perform within the oral environment, ultimately informing clinical decision-making for optimal patient outcomes in dental practice.
